# Quarterly Atlas of Dermatology

**Published:** 1895-11

**Authors:** 


Quarterly Atlas of Dermatology: An Illustrated Quarterly Journal of Skin and Venereal Diseases. Edited by A. H. Ohmann Dumesnil, A. M., M. D., Professor of Dermatology and Syphilology in the Marion-Sims College of Medicine of St. Louis. Published by the Quarterly Atlas Co., 1 North Broadway, St. Louis, Mo., U. S. A. Subscription price, $1.00 per annum.

The October number of this important publication is before the editor. It contains articles on Ecthyma, Cornu Unguale, Subacute Eczema, Porrigo è Pediculus, Varioliform, Syphilide, and Pustulo Crustaceous Syphilide. Every one of these articles is illustrated by a photographic plate. It is certainly a very desirable journal for the homœopathic physician. The treatment, however, is of the orthodox character. This, however, need not trouble the homœopathist. What he wants to get is a clear idea of the different skin diseases as found in these descriptions and illustrations.

